# Research and Remedies

**Published:** 1915-08-28

**Authors:** 


### RESEARCH AND REMEDIES.

### Acetone and Vomiting.

Now and again one meets with the case of a child who has recurrent attacks of vomiting associated with acidosis, the latter announced by the odour of acetone in the breath. This latter may be so pronounced that it is appreciated by anyone in the sick room and inevitably suggests an advanced case of diabetes. The explanation of these cases is very difficult, but the condition is generally regarded as due to some disturbance of the hepatic functions. The preventive treatment is to avoid excess of carbo-hydrate foods, and to see that the bowels are kept on the free side by an occasional dose of calomel. Fenner, writing on the treatment of the attack, insists on the necessity of treating the vomiting rather than the acidosis. At the outset fractional doses of calomel should be tried, though even these may be rejected. To order, as is frequently done, large doses of sodium bicarbonate with the idea of neutralising the acid is worse than useless. The sovereign remedy, he says, is morphine. It may be given hypodermically, or, in the milder cases, by mouth, in very small doses, and combined with a minute dose of cocaine and a few grains of magnesia in a little chloroform water or plain water. The thirst, which is a prominent symptom, can be met by saline injections given by the " drip " method per rectum, and the drowsiness produced by the morphine favours this procedure. When the vomiting has ceased and the bowel has resumed its function some nourishment may be tried by the mouth, and small quantities of dilute condensed milk or malted milk may be tried.—*New Orleans Medical and Surgical Journal.*

### The Luetin Test for Syphilis.

Luetin consists of several pure strains of the *Spirochæta pallida* killed by heat and containing tricresol as a preservative. It is injected intradermically, not hypodermically, and in order that it may be introduced actually between the layers of the skin a very fine needle with a short bevel is employed. The usual steps for sterilising the skin must, of course, be adopted. If the test is positive there appears at the site of puncture and within forty-eight hours an indurated, inflamed deeply seated nodule, varying in size from a pea to a hazel-nut, or even larger. The point of importance is the persistence or disappearance of this nodule. If it lasts less than a week it is not sufficient as a basis for a confident diagnosis. Sometimes, however, subsidence is followed after a week or more by reappearance (" delayed reaction "), and this is taken as a sign of late or latent lues. Noting these two possibilities, it is the persistence of the induration for a week or longer which is the mark of a positive result. The nodule varies not only in size, but also in the degree of inflammation with which it is accompanied; sometimes there is a pustule-like point at its summit. It gradually subsides, but more or less pigmentation and some thickening may persist indefinitely. The conclusions formulated by Noguchi are as follows: In early syphilis the reaction occurs in less than one-third of the cases and is usually mild; in secondary syphilis in less than one-half there is a mild reaction; but in late stages of the disease a positive result is obtained in 80 per cent. of the cases, and in the congenital form of the disease the same result is obtained in over 70 per cent. In the so-called parasyphilitic cases there are no constant findings. The reaction does not always run parallel with the Wassermann test, though frequently when one is positive, so is the other, but treated cases often show negative Wassermann and positive luetin reactions.—*California State Journal of Medicine.*

### Diet in Advanced Heart Disease.

The scheme proposed by Karell, a Russian physician, in uncompensated heart disease, especially when attended by dropsies, was a restriction of the diet to skimmed milk. This proposal may be adopted in a somewhat modified form as follows: For the first five days allow 800 to 1,000 c.c. of milk daily, given in small amounts at frequent intervals, all other fluids and foods being forbidden. If thirst is troublesome a little lemon juice may be given, or, better still, small amounts of morphine every few hours. After five days the amount of milk may be somewhat increased, and toast with unsalted butter and rice may be added; and after a further period of five days a still larger liberality may be practised. Evidences of improvement are greater comfort and less dyspnœa in the patient, better quality of pulse, while there is now a response to digitalis, which at an earlier stage may have proved useless. Another favourable sign is increased diuresis, and, indeed, this may be said to be the touch-stone of the treatment. Albuminuria, if due to congestion of the kidney, will lessen or disappear. On the other hand, its persistence suggests organic renal disease, and in these circumstances the Karell regimen is contraindicated. There is a special opportunity for this method of treatment in cases in which the functions of the stomach are disturbed and it is impossible to continue the use of digitalis. When this is the case digitalis should be resumed as soon as possible, and in many instances the addition of small doses of morphine is a valuable help. A minimal dose, say, 1/24 grain, every three hours, often has a very good effect, and it is best to order it by the mouth. The writer's emphasis as to the value of morphine is very much to the point. For some reason or other many practitioners are afraid to use this medicine in heart disease. It is well to remove this fear, for the soothing influence of morphine on the nervous system and on the patient's general condition is often most helpful.—*Dr. Taussig, in the " Interstate Medical Journal."*

